# Supplementary material for: The use of precision diagnostics for monogenic diabetes: a systematic review and expert opinion
Source: Commun Med (Lond). 2023 Oct 5;3:136. doi: 10.1038/s43856-023-00369-8 (PMC10550998; doi:10.1038/s43856-023-00369-8)
Supplement: Supplementary file 1 — Supplementary Information [file 43856_2023_369_MOESM1_ESM.pdf]

SUPPLEMENTARY ONLINE INFORMATION

The use of Precision Diagnostics for Monogenic Diabetes: a Systematic Review and Expert Opinion

Rinki Murphy, Kevin Colclough, Toni I. Pollin, Jennifer M. Ikle, Pernille Svalastoga, Kristin A. Maloney, Cécile Saint-Martin, Janne Molnes, ADA/EASD PMDI, Shivani Misra, Ingvild Aukrust, Elisa de Franco, Sarah E. Flanagan, Pål R. Njølstad, Liana K. Billings, Katharine R Owen, Anna L Gloyn

Table of contents **Page**

Supplementary Table 1	1
Supplementary Table 2	8
Supplementary Figure 1	9
Supplementary Figure 2	10
Supplementary Table 3	12
Description of Supplementary Data Sets	18

Supplementary Table 1: Search strategy using PubMed & Embase

**Question 1: Who should you test for monogenic diabetes**	**Number of References**
**Query**	
**#1**<br>Diabetes Mellitus, Permanent Neonatal" [Supplementary Concept]) OR ("Maturity-Onset Diabetes Of The Young, Type 9" [Supplementary Concept] OR "Maturity-Onset Diabetes of the Young, Type 7" [Supplementary Concept] OR "MODY, Type 6" [Supplementary Concept] OR "Maturity-Onset Diabetes of the Young, Type 8, with Exocrine Dysfunction" [Supplementary Concept] OR "Maturity-Onset Diabetes of the Young, Type 1" [Supplementary Concept] OR "Maturity-Onset Diabetes of the Young, Type 2" [Supplementary Concept] OR "Maturity-Onset Diabetes of the Young, Type 3" [Supplementary Concept] OR "Maturity-Onset Diabetes of the Young, Type 4" [Supplementary Concept])) OR ("Lipodystrophy, Congenital Generalized"[Mesh] OR "Diabetes Mellitus, Congenital Autoimmune" [Supplementary Concept])) OR "Lipodystrophy"[Mesh]) OR "Pancreatic Agenesis, Congenital" [Supplementary Concept]) OR "Pancreatic Hypoplasia, Congenital, with Diabetes Mellitus and Congenital Heart Disease" [Supplementary Concept]) OR "Donohue Syndrome"[Mesh]) OR "Mason-Type Diabetes" [Supplementary Concept]) OR "Lipodystrophy, Familial Partial"[Mesh]) OR "Renal cysts and diabetes syndrome" [Supplementary Concept]) OR ("Diabetes Mellitus, Transient Neonatal, 2" [Supplementary Concept] OR "Diabetes Mellitus, Transient Neonatal, 3" [Supplementary Concept] OR "Diabetes Mellitus, Transient Neonatal, 1" [Supplementary Concept] OR "6q24-Related Transient Neonatal Diabetes Mellitus" [Supplementary Concept])) OR "Noninsulin-dependent diabetes mellitus with deafness" [Supplementary Concept] Filters: English	**4731**
**#2**<br>"monogenic diabetes"[Title/Abstract] OR "neonatal diabetes"[Title/Abstract] OR "maturity onset diabetes of the young"[Title/Abstract] OR "maturity-onset diabetes of the young"[Title/Abstract] OR "infancy onset diabetes"[Title/Abstract] OR "infancy-onset diabetes"[Title/Abstract] OR "congenital diabetes"[Title/Abstract] OR TNDM OR PNDM* OR "early onset diabetes"[Title/Abstract] OR "early-onset diabetes"[Title/Abstract] OR "syndromic diabetes"[Title/Abstract] OR lipodystroph* OR "mitochondrial diabetes"[Title/Abstract] OR "pancreatic agenesis"[Title/Abstract] OR "pancreatic hypoplasia"[Title/Abstract] OR leprechaun* OR "donohue syndrome"[Title/Abstract] OR "rabson mendenhall"[Title/Abstract] OR rabson-mendenhall OR dunnigan OR "wolfram syndrome"[Title/Abstract] OR RCAD OR "renal cysts and diabetes"[Title/Abstract] OR "chromosome 17q12 deletion syndrome"[Title/Abstract] OR "chromosome-17q12 deletion syndrome"[Title/Abstract] OR "mason type diabetes"[Title/Abstract] OR "mason-type diabetes"[Title/Abstract] OR "transient neonatal diabetes"[Title/Abstract] OR "permanent neonatal diabetes"[Title/Abstract] OR maternally inherited diabetes and deafness OR "M.3243A>G mutation"[Title/Abstract] OR "mitochondrial tRNA"[Title/Abstract] OR "MODY" Filters: English	**14256**
**#3**<br>**#1 OR #2**	**14295**

**#4**<br>“Hepatocyte Nuclear Factor 1-beta"[Mesh] OR "Hepatocyte Nuclear Factor 1-alpha"[Mesh] OR "Hepatocyte Nuclear Factor 4"[Mesh] Filters: English	**3585**
**#5**<br>HNF1A OR HNF1-A OR "hepatocyte nuclear factor 1"[Title/Abstract] OR "hepatocyte nuclear factor 1a"[Title/Abstract] OR "hepatocyte nuclear factor 1 alpha"[Title/Abstract] OR "HNF1 alpha"[Title/Abstract] OR HNF1alpha OR HNF4A OR HNF4-A OR HNF-4A OR "hepatocyte nuclear factor 4"[Title/Abstract] OR "hepatocyte nuclear factor 4a"[Title/Abstract] OR "hepatocyte nuclear factor 4 alpha"[Title/Abstract] OR HNF4alpha OR GCK OR HNF1B OR HNF1-b OR HNF-1B OR "hepatocyte nuclear factor 1b"[Title/Abstract] OR "hepatocyte nuclear factor 1 beta"[Title/Abstract] OR HNF1beta OR "HNF-1 beta"[Title/Abstract] OR "INS gene"[Title/Abstract] OR "INS mutation"[Title/Abstract] OR KCNJ11[Title/Abstract] OR ABCC8[Title/Abstract] OR PDX1[Title/Abstract] OR CEL-MODY [Title/Abstract] OR "CEL gene"[Title/Abstract] OR "CEL mutation"[Title/Abstract] OR "caroboxyl ester lipase"[Title/Abstract] OR NEUROD1[Title/Abstract] OR WFS1[Title/Abstract]	**11142**
**#6**<br>(INSR[Title/Abstract]) AND (diabetes[Title/Abstract]) Filters: English	**273**
**#7**<br>(LMNA[Title/Abstract] OR PPARG[Title/Abstract] OR PLIN1[Title/Abstract]) AND (lipodystrophy[Title/Abstract]) Filters: English	**347**
**#8**<br>#4 OR #5 OR #6 OR #7	**11988**
**#9**<br>#3 OR #8	**23982**
**#10**<br>"Diagnosis"[Mesh] Filters: English	**7662064**
**#11**<br>"Phenotype"[Mesh] Filters:English	**307182**
**#12**<br>Neonatal Screening[Mesh] Filters:English	**9677**
**#13**<br>"etiology" [Subheading] Filters:English	**8144644**

**#14**<br>Biomarkers[MeSH] Filters:English	**760545**
**#15**<br>#10 OR #11 OR #12 OR #13 OR #14	**7868628**
**#16**<br>diagnosis[Title/Abstract] Filters: English	**1367001**
**#17**<br>"clinical characterist*"[Title/Abstract] Filters: English	**72805**
**#18**<br>"identif*"[Title/Abstract] Filters:English	**3418989**
**#19**<br>phenotyp*[Title/Abstract] Filters: English	**613305**
**#20**<br>"risk score*"[Title/Abstract] Filters: English	**25785**
**#21**<br>"screening pathway*"[Title/Abstract] Filters: English	**127**
**#22**<br>etiolog*[Title/Abstract] Filters: English	**258874**
**#23**<br>"systematic assessment"[Title/Abstract] Filters: English	**2720**
**#24**<br>biomarker*[Title/Abstract] Filters: English	**320083**
**#25**<br>"clinical suspicion"[Title/Abstract] Filters: English	**12335**
**#26**<br>#16 OR #17 OR #18 OR #19 OR #20 OR #21 OR #22 OR #23 OR #24 OR #25	**5290922**

**#27**<br>#15 OR #26	**7868578**
**#28**<br>#9 AND #27	**7487**
**#29**<br>#28 Filters: English, Humans	**6119**
1990-2000: 626<br>2001-2010: 2138<br>2011-2021: 3025	
**Question 2 How should you test someone for monogenic diabetes**	
**#30**<br>(((("High-Throughput Nucleotide Sequencing"[Mesh]) OR "Whole Exome Sequencing"[Mesh]) OR "Multiplex Polymerase Chain Reaction"[Mesh]) OR "Real-Time Polymerase Chain Reaction"[Mesh]) OR "Chromatography, High Pressure Liquid"[Mesh]) OR "Polymorphism, Restriction Fragment Length"[Mesh] Filters: Humans, English	**156758**
**#31**<br>"next generation sequencing"[Title/Abstract] OR "next-generation sequencing"[Title/Abstract] OR "sanger sequencing"[Title/Abstract] OR "dna sequencing"[Title/Abstract] OR "multigene panel"[Title/Abstract] OR "genetic testing"[Title/Abstract] OR "genetic screening"[Title/Abstract] OR "dna pooling"[Title/Abstract] OR "high-throughput sequencing"[Title/Abstract] OR "high throughput sequencing"[Title/Abstract] OR "targeted sequencing"[Title/Abstract] OR "target region capture sequencing"[Title/Abstract] OR "exome sequencing"[Title/Abstract] OR "genome sequencing"[Title/Abstract] OR mlpa OR "dosage analysis"[Title/Abstract] OR "cnv detection"[Title/Abstract] OR "cnv analysis"[Title/Abstract] OR "allele specific pcr"[Title/Abstract] OR "real-time pcr"[Title/Abstract] OR "fluorescent pcr"[Title/Abstract] OR taqman OR sscp OR hplc OR rflp OR "chip n3 genotyping"[Title/Abstract] OR "whole exome sequencing"[Title/Abstract] OR "multiplex ligation dependent probe amplification"[Title/Abstract] OR "allele specific polymerase chain reaction"[Title/Abstract] OR "real time polymerase chain reaction"[Title/Abstract] OR "single strand conformation polymorphism"[Title/Abstract] OR "high performance liquid chromatography"[Title/Abstract] OR "restriction fragment length polymorphism"[Title/Abstract] Filters: Humans, English<br>=304221	**304221**
**#32**<br>#30 OR #31	**335251**
**#33**<br>#9 AND #32	**1460**

Paper per decade	1990-2000: 114<br>2001-2010: 301<br>2011-2021: 1041
**Question 6 What are the next steps after a diagnosis of monogenic diabetes**	
#91<br>("Genetic Counseling"[Mesh]) OR "Cell-Free Nucleic Acids"[Mesh] Filters: Humans, English	16553
#92<br>"cascade testing"[Title/Abstract] OR "cascade screening"[Title/Abstract] OR "genetic counselling"[Title/Abstract] OR "cell-free dna"[Title/Abstract] OR "cell free dna"[Title/Abstract] OR "noninvasive prenatal testing"[Title/Abstract] OR "non-invasive prenatal testing"[Title/Abstract] Filters: Humans, English	**8338**
#93<br>#91 OR #92	21814
#94<br>#9 AND #93	**61**
#95<br>"Penetrance"[Mesh] OR "Oceanic Ancestry Group"[Mesh] OR "American Native Continental Ancestry Group"[Mesh] OR "Asian Continental Ancestry Group"[Mesh] OR "European Continental Ancestry Group"[Mesh] OR "African Continental Ancestry Group"[Mesh] OR "Economic evaluation"[Mesh] OR "Continental Population Groups"[Mesh] Filters: Humans, English	**223571**
#96<br>variant[Title/Abstract]) AND (unknown[Title/Abstract] OR significan*[Title/Abstract] OR ancestry group[Title/Abstract] OR penetrance[Title/Abstract] OR economic evaluation[Title/Abstract]) Filters: Humans, English	**4866**
#97<br>#95 OR #96	**2601031**
#98<br>#9 AND #97	**844**
Papers per decade	*1990-2000: 87*<br>*2001-2010: 308*<br>*2011-2020: 492*
**Question 7 What are the current challenges for the field in precision diagnostics?**	
**#26**<br>'variant'/exp OR 'ancestry group'/exp OR 'penetrance'/exp OR 'economic evaluation'/exp	992252
**#27**<br>('variant of unknown significance':ab,ti OR 'variant n3':ab,ti) AND (unknown:ab,ti OR significan*:ab,ti) OR 'ancestry group':ab,ti OR penetrance:ab,ti OR 'economic evaluation':ab,ti	33291

**#28** #26 OR #27	992252
Combined Search<br>#35 #7 AND #28<br>2230 records<br>AND [embase]/lim NOT ([embase]/lim AND [medline]/lim)<br>1004 records<br>Filter Human, English<br>859 records<br>NOT ‘conference abstract’:it	**201**
Papers per decade	*1990-2000: 4*<br>*2001-2010: 32*<br>*2011-2020:165*

Supplementary Table 2 PICOTS

	**Diagnostic validity**
**Population**	Patients with diabetes
**Intervention**	Genetic testing for monogenic diabetes (using at least sequencing of a single gene if not multiple genes or whole exome or whole genome)
**Comparison**	Unselected vs selected cases using various clinical or biomarker criteria to increase yield of monogenic diabetes
**Outcomes**	· Diagnostic yield (fraction and percentage)<br>· Sensitivity/specificity (if thresholded)<br>· AUROC (if not thresholded)<br>· PPV/NPV
**Timing**	n/a
**Setting**	n/a

Supplementary Figure 1

A

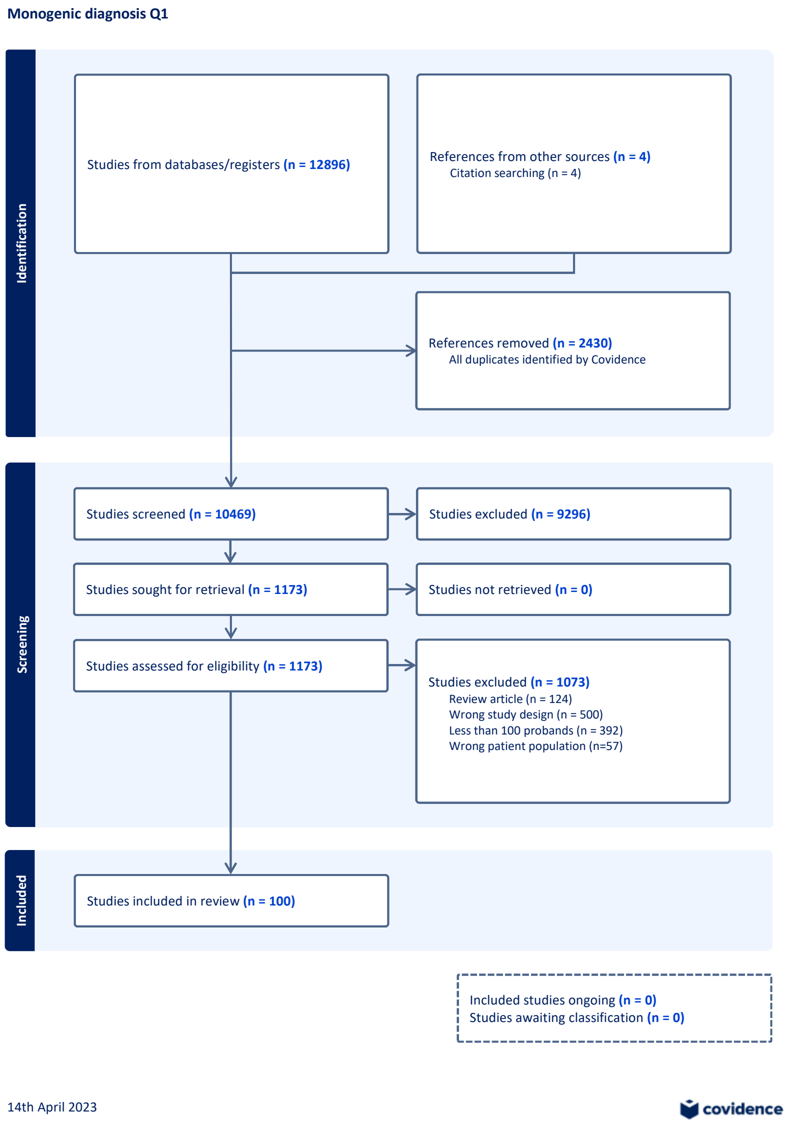

B

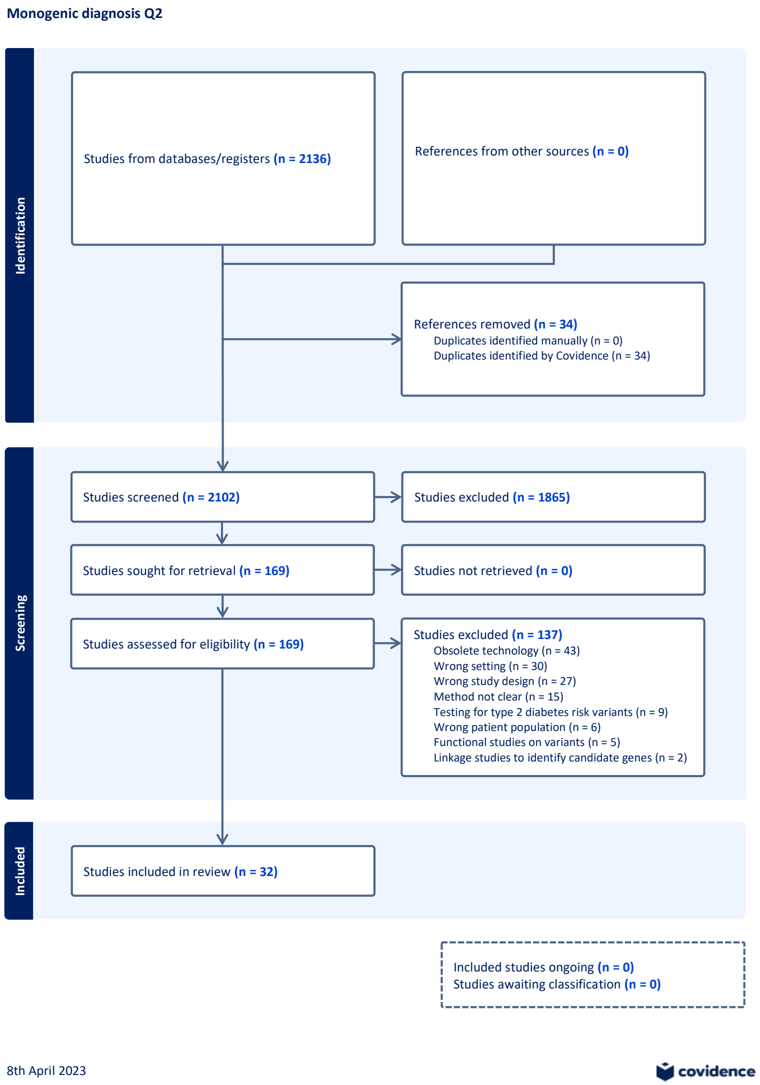

Supplementary Figure 2. Critical appraisal 10-item checklist definitions, criteria for assigning levels of evidence and grades of recommendations on how to select probands with diabetes for monogenic diabetes genetic testing

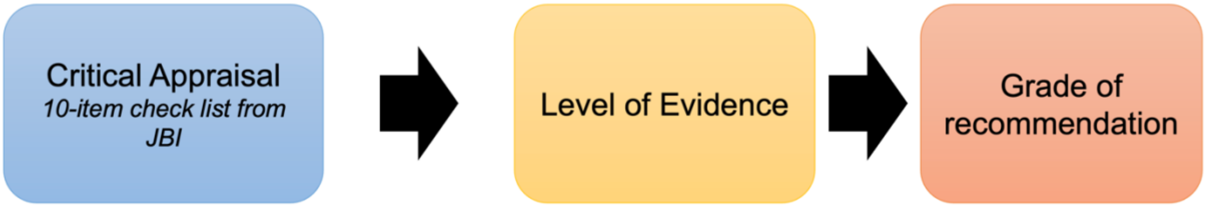

CRITERIA FOR ASSIGNING LEVELS OF EVIDENCE TO THE PUBLISHED STUDIES OF DIAGNOSIS

Level	Criteria
Level 1	(a) independent interpretation of triage test results (without knowledge of the result of the diagnostic genetic test) – *item 4 from JBI check list [assumed to be uniformly positive]*<br>(b) independent interpretation of the diagnostic genetic test (without knowledge of the triage test result) – *item 7 from JBI check list [assumed to be uniformly positive]*<br>(c) selection of people suspected (but not known to have the disorder) - *item 2 from JBI check list*<br>(d) reproducible description of the triage test and diagnostic genetic test - *item 5 and 6 from the JBI checklist*<br>(d) at least 50 patients with and 50 patients without the disorder
Level 2	Meets 4 of the Level 1 criteria
Level 3	Meets 3 of the Level 1 criteria
Level 4	Meets 1 or 2 of the Level 1 criteria

CRITERIA FOR ASSIGNING GRADES OF RECOMMENDATIONS FOR CLINICAL PRACTICE

Grade	Criteria
Grade A	The best evidence was at Level 1
Grade B	The best evidence was at Level 2
Grade C	The best evidence was at Level 3
Grade D	The best evidence was at Level 4 or consensus

JBI CHECK LIST FOR EVALUATING DIAGNOSTIC ACCURACY

PATIENT SELECTION

1. Was a consecutive or random sample of patients enrolled?

Studies should state or describe their method of enrolment. If it is claimed that a random sample was chosen the method of randomization should be stated (and appropriate) for 'yes'. It is 'Yes' if studies do not say 'consecutive' but instead describe consecutive enrolment.

2. Was a case control design avoided?

If a study design involves recruiting participants who are already known by other means to have the diagnosis of interest (MD cases) and is investigating whether the test of interest correctly identifies them as such, the answer is 'No'.

3. Did the study avoid inappropriate exclusions?

If patients are excluded for reasons that would likely influence the conduct, interpretation or results of the index test, this may bias the results. Examples include: excluding patients on which the index test is difficult to conduct, excluding patients with borderline results, excluding patients with clear clinical indicators of the diagnosis of interest.

INDEX TEST

4. Were the index test results interpreted without knowledge of the results of the reference standard?

The results of the index test (biomarker, clinical signs) should be interpreted by someone who is blind to the results of the reference test (MD genetic result). The reference test may not have been conducted at the point that the index test is carried out, if so the answer to this question will be 'Yes'. Only if the person who interprets the index test also interpreted the reference genetic test will this question be answered 'No' (unlikely to apply in MD context).

5. If a threshold was used, was it pre-specified?

Diagnostic thresholds may be chosen based on what gives the optimum accuracy from the data, or they may be pre-specified.

REFERENCE TEST

6. Is the reference standard likely to correctly classify the target condition?

The reference test should be the gold standard for the diagnosis of monogenic diabetes and should include at least a 6 gene panel with optimal gene variant curation.

7. Were the reference standard results interpreted without knowledge of the results of the index test?

Similar to criteria 4: The results of the reference test (genetic result) should be interpreted by someone who is blind to the results of the index test (clinical/biomarker), even though ACMG curation requires some knowledge of clinical features. It is only if the person who interprets the reference test (genetic test) also interpreted the index test (clinical features) then this question will be answered 'No' – so we are unlikely to answer No for our studies.

8. Was there an appropriate interval between index test and reference standard?

The index test and the reference test should be carried out close enough together that the status of the patient could not have meaningfully changed. For our context this will be N/A

9. Did all patients receive the same reference standard?

The reference standard (genetic test) by which patients are classified as having or not having the condition of interest (MD) should be the same for all patients – answer 'Yes'. But if the diagnostic approach is that the results of the index test (eg: antibody negativity or certain # of clinical signs) influences how or whether the reference test is used (i.e. genetic test is only offered to antibody negative individuals), then an apparent false negative may be detected so this may result in biased estimates of sensitivity and specificity. In this case the answer should be 'No'.

10. Were all patients included in the analysis?

Losses to follow up should be explained and their cause and frequency should be considered in whether they are likely to have had an effect on the results.

CRITERIA FOR ASSIGNING LEVELS OF EVIDENCE TO THE PUBLISHED STUDIES OF DIAGNOSIS

Level	Criteria
Level 1	(a) independent interpretation of triage test results (without knowledge of the result of the diagnostic genetic test) – *item 4 from JBI check list [assumed to be uniformly positive]*<br>(b) independent interpretation of the diagnostic genetic test (without knowledge of the triage test result) – *item 7 from JBI check list [assumed to be uniformly positive]*<br>(c) selection of people suspected (but not known to have the disorder) - *item 2 from JBI check list*<br>(d) reproducible description of the triage test and diagnostic genetic test - *item 5 and 6 from the JBI checklist*<br>(d) at least 50 patients with and 50 patients without the disorder
Level 2	Meets 4 of the Level 1 criteria
Level 3	Meets 3 of the Level 1 criteria
Level 4	Meets 1 or 2 of the Level 1 criteria

Supplementary Table 3: Complete set of papers extracted for question 2. How to test for monogenic diabetes

Study Details	Individuals tested	Test methodology and number of different genes tested	Genes analysed	Individuals diagnosed, diagnostic yield and number of different genetic subtypes diagnosed	Number of patients diagnosed by subtype:
Abbasi, 2018 Iran [1]	60 neonates with NDM	Sanger sequencing; 4	*ABCC8; EIF2AK3; INS; KCNJ11*	11 (18%); all *EIF2AK3*	11 *EIF2AK3*
Al-Senani, 2018 Oman [2]	18 neonates with NDM	tNGS (RNA baits) and MS-PCR; 24	6q24*; ABCC8; EIF2AK3; FOXP3; GATA4; GATA6; GCK; GLIS3; HNF1B; INS; KCNJ11; IER3IP1; IL2RA; LRBA; NEUROD1; NEUROG3; NKX2-2; PDX1; PTF1A; RFX6; SLC2A2; SLC19A2; STAT3; WFS1; ZFP57*	9 (50%); 2	5 *GCK*, 1 *KCNJ11*
Al-Kandari, 2021 Kuwait [3]	31 children with suspected MODY	MLPA and targeted exome sequencing (gene panel); 22	*ABCC8; CISD2; CEL; GATA6; GCK; HNF1A; HNF4A; HNF1B; INS; INSR; KCNJ11; LMNA; NEUROD1;* m.3243A>G*; PAX6; PDX1; PLIN1; PPARG; RFX6; WFS1; ZFP57*	7 (23%); 5	1 *GCK*, 2 *HNF1A*, 1 *HNF4A*, 2 *HNF1B*, 1 *PDX*
Al-Khawaga, 2019 Qatar [4]	7 neonates with NDM	WES (gene agnostic), WGS (gene agnostic) and CNV analysis as part of tNGS/exome/genome sequencing; Not Stated	*Whole exome; Whole genome*	7 (100%); 6	1 *GCK*, 1 *HNF1B*, 2 *INS*, 1 *EIF2AK3*, 1 *PTF1A*
Alkorta-Aranburu, 2014 USA [5]	44 children and adults with suspected MODY and 32 with neonates with NDM	WES (gene agnostic), WGS (gene agnostic) and CNV analysis as part of tNGS/exome/genome sequencing; Not Stated	*ABCC8; AKT2; ALMS1; BLK; CISD2; CEL; CP; DCAF17; EIF2AK3; FOXP3; GATA6; GCK; GLIS3; GLUD1; HADH; HNF1A; HNF4A; HNF1B; IER3IP1; INS; INSR; KCNJ11; NEUROD1; NEUROG3; PAX4; PAX6; PDX1; PTF1A; RFX6; SLC2A2; SLC19A2; SLC29A3; TBC1D4; WFS1; ZFP57*	12 with MODY (27%); 2 and 7 with NDM (22%); 5	11 *GCK*, 2 *HNF1A*, 1 *ABCC8*, 2 *KCNJ11*, 2 *INS*, 1 *EIF2AK3*
Alkorta-Aranburu, 2016 USA [6]	22 neonates with NDM	MS-MLPA and tNGS (RNA baits); 11	6q24*; ABCC8; EIF2AK3; FOXP3; GATA4; GCK; INS; KCNJ11; MNX1; NKX2-2; PDX1; ZFP57*	14 (64%), 5	2 *ABCC8*, 2 *KCNJ11*, 1 *INS*, 1 *FOXP3*, 1 6q24
Ang, 2016 Singapore [7]	84 adults with suspected MODY	tNGS (PCR using Ion AmpliSeq) and Genotyping (TaqMan); 16	*ABCC8; BLK; CEL; GCK; HNF1A; HNF4A; HNF1B; INS; INSR; KCNJ11; KLF11; LMNA; NEUROD1;* m.3243A>G*; PAX4; PDX1; PPARG*	13 (16%), 7	1 *GCK*, 4 *HNF1A*, 1 *HNF4A*, 1 *ABCC8*, 1 *KCNJ11*, 2 m.3243A>G
Anik, 2015 Turkey [8]	42 children with suspected MODY	tNGS (PCR using Illumina Nextera XT); 11	*BLK; CEL; GCK; HNF1A; HNF4A; HNF1B; INS; KLF11; NEUROD1; PAX4; PDX1*	12 (29%), 5	8 *GCK*, 1 *HNF1A*, 1 *HNF1B*, 1 *PDX1*, 1 *BLK*
Antosik, 2016 Poland [9]	1 neonate with NDM	tNGS (RNA baits); 1	*GCK*	1 (100%), 1	1 *GCK*

Ateş, 2021 Turkey [10]	182 adults with suspected MODY	tNGS (PCR using Agilent MODY-MASTR assay); 7	*ABCC8; GCK; HNF1A; HNF4A; HNF1B; INS; KCNJ11*	30 (17%), 6	10 *GCK*, 9 *HNF1A*, 2 *HNF4A*, 2 *HNF1B*, 6 *ABCC8*, 1 *KCNJ11*
Bansal, 2017 USA [11]	4016 adults with type 2 DM	tNGS (RNA baits); 22	*ABCC8; BLK; CEL; EIF2AK3; GATA6; GCK; GLIS3; HNF1A; HNF4A; HNF1B; INS; KCNJ11; KLF11; NEUROD1; NEUROG3; PAX4; PAX6; PDX1; PPARG; RFX6; SLC19A2; WFS1*	53 (1%), 8	21 *GCK*, 17 *HNF1A*, 5 *HNF4A*, 1 *HNF1B*, 6 *ABCC8*, 1 *INS*, 1 *WFS1*, 1 *PPARG*
Berberich, 2021 Canada [12]	57 children and adults wth suspected MODY	tNGS (DNA baits) and CNV analysis as part of tNGS; 14	*ABCC8; CEL; GCK; HNF1A; HNF4A; HNF1B; INS; INSR; KCNJ11; LMNA; NEUROD1; PDX1; PPARG; RFX6*	3 (5%), 1	3 *HNF1B*
Carette, 2010 France [13]	84 children and adults wth suspected MODY	Sanger sequencing and MLPA; 2	*HNF1A; HNF4A*	8 (10%), 2	2 *HNF1A*, 6 *HNF4A*
Caswell, 2020 UK [14]	33 at-risk pregnancies	Droplet digital PCR; 1	*GCK*	21 (64%), 1	21 *GCK*
Chambers, 2016 USA [15]	97 children and adults wth suspected MODY	Sanger sequencing; 5	*GCK; HNF1A; HNF4A; HNF1B; PDX1*	20 (21%), 3	8 *GCK*, 9 *HNF1A*, 3 *HNF4A*
Colclough, 2022 UK [16]	1280 children and adults wth suspected MODY	tNGS (RNA baits) and CNV analysis as part of tNGS; 27	*ABCC8; CISD2; CEL; GATA4; GATA6; GCK; HNF1A; HNF4A; HNF1B; INS; INSR; KCNJ11; LMNA; NEUROD1;* m.3243A>G; *PAX6; PCBD1; PDX1; PLIN1; POLD1; PPARG; RFX6; SLC29A3; TRMT10A; WFS1; ZBTB20; ZFP57*	297 (23%), 17	66 *GCK*, 98 *HNF1A*, 42 *HNF4A*, 18 *HNF1B*, 11 *ABCC8*, 5 *KCNJ11*, 6 *INS*, 8 *RFX6*, 3 *NEUROD1*, 2 *PDX1*, 24 m.3243A>G, 6 *WFS1*, 4 *INSR*, 1 *PPARG*, 1 *TRMT10A*, 1 *SLC29A3*, 1 *GATA6*
De Franco, 2015 UK [17]	1020 neonates with NDM	Sanger sequencing, MS-MLPA) and tNGS (RNA baits) and CNV analysis as part of tNGS; 23	6q24*; ABCC8; EIF2AK3; FOXP3; GATA4; GATA6; GCK; GLIS3; HNF1B; IER3IP1; INS; KCNJ11; IER3IP1; MNX1; NEUROD1; NEUROG3; NKX2-2; PDX1; PTF1A; RFX6; SLC2A2; SLC19A2; ZFP57*	840 (82%), 22	30 *GCK*, 2 *HNF1B*, 150 *ABCC8*, 240 *KCNJ11*, 110 *INS*, 1 *RFX6*, 3 *NEUROD1*, 6 *PDX1*, 76 *EIF2AK3*, 22 *PTF1A*, 14 *FOXP3*, 113 6q24, 7 *SLC19A2*, 4 *GATA6*, 9 *GLIS3*, 1 *IER3IP1*, 1 *MNX1*, 1 *NEUROG3*, 2 *NKX2-2*, 6 *SLC2A2*, 12 *ZFP57*
Donath, 2019 France [18]	1564 children and adults wth suspected MODY	MLPA and tNGS (PCR using Agilent MODY-MASTR assay); 7	*ABCC8; GCK; HNF1A; HNF4A; HNF1B; INS; KCNJ11*	254 (16%), 7	109 *GCK*, 82 *HNF1A*, 25 *HNF4A*, 15 *HNF1B*, 8 *ABCC8*, 3 *KCNJ11*, 7 *INS*,
Ellard, 2013 UK [19]	33 children with suspected MODY and 49 neonates with NDM	tNGS (RNA baits) and CNV analysis as part of tNGS; 29	*ABCC8; BLK; CEL; EIF2AK3; FOXP3; GATA4; GATA6; GCK; GLIS3; HNF1A; HNF4A; HNF1B; INS; KCNJ11; KLF11; IER3IP1; LMNA; NEUROD1; NEUROG3;* m.3243A>G; *PAX6; PDX1; PPARG; PTF1A; RFX6; SLC2A2; SLC19A2; WFS1; ZFP57*	5 with MODY (15%), 4 and 9 with NDM (18%), 6	3 *GCK*, 1 *HNF4A*, 1 *HNF1B*, 1 *ABCC8*, 1 *PDX1*, 2 m.3243A>G, 1 *EIF2AK3*, 2 *SLC19A2*, 2 *GATA6*

Ellard, 2007 UK [20]	90 children and adults wth suspected MODY	MLPA; 3	*GCK; HNF1A; HNF4A*	6 (7%), 2	1 *GCK*, 5 *HNF1A*
Johansson, 2012 Norway [21]	9 children and adults wth suspected MODY	targeted exome sequencing (gene panel); 109	*ABCC8; BLK; CISD2; CEL; EIF2AK3; GATA4; GATA6; GCK; GLIS3; HADH; HNF1A; HNF4A; HNF1B; INSR; KCNJ11; KLF11; LMNA; MNX1; NEUROD1; NEUROG3; NKX2-2; PAX4; PAX6; PDX1; POLD1; PPARG; PTF1A; RFX6; SLC2A2; WFS1*	3 (33%), 3	1 *HNF4A*, 1 *ABCC8*, 1 *PPARG*
Laimon, 2021 Egypt [22]	26 neonates with NDM	Sanger sequencing, tNGS (RNA baits) and CNV analysis as part of tNGS; 22	*ABCC8; EIF2AK3; FOXP3; GATA4; GATA6; GCK; GLIS3; HNF1B; INS; KCNJ11; IER3IP1; NEUROD1; NEUROG3; NKX2-2; PDX1; PTF1A; RFX6; SLC2A2; SLC19A2; STAT3; WFS1*	14 (54%), 7	1 *GCK*, 2 *ABCC8*, 2 *KCNJ11*, 4 *INS*, 3 *EIF2AK3*, 1 *SLC19A2*, 1 *INSR*
Patel, 2022 Greece [23]	236 children with suspected MODY	tNGS (RNA baits) and CNV analysis as part of tNGS; 51	*ABCC8; AGPAT2; AKT2; APPL1; BSCL2; CISD2; CEL; COQ2; CTLA4; EIF2AK3; FOXP3; GATA4; GATA6; GCK; GLIS3; HNF1A; HNF4A; HNF1B; IER3IP1; IL2RA; INS; INSR; ITCH; KCNJ11; LMNA; LRBA; MNX1; NEUROD1; NEUROG3; NKX2-2;* m.3243A>G*; PAX6; PCBD1; PDX1; PIK3R1; PLIN1; POLD1; PPARG; PTF1A; RFX6; SIRT1; SLC2A2; SLC19A2; SLC29A3; STAT1; STAT3; STAT5B; TRMT10A; WFS1; ZFP57*	34 (14%), 12	11 *GCK*, 3 *HNF1A*, 1 *HNF4A*, 2 *HNF1B*, 1 *KCNJ11*, 2 *INS*, 1 m.3243A>G, 1 *PTF1A*, 7 *WFS1*, 3 *SLC19A2*, 1 *SLC29A3*, 1 *TRMT10A*
Patouni, 2021 Greece [24]	1 child with type 1 DM	Sanger sequencing and MLPA; 3	*GCK; HNF1A; HNF1B*	1 (100%), 2	1 *HNF1A*, 1 *HNF1B*
Pruhova, 2010 Czech Republic [25]	140 children and adults wth suspected MODY	Sanger sequencing and MLPA; 1	*GCK*	103 (74%), 1	103 *GCK*
Saint-Martin, 2022 France [26]	1676 children and adults wth suspected MODY	tNGS (RNA baits) and CNV analysis as part of tNGS; 18	*ABCC8; CISD2; GATA4; GATA6; GCK; HNF1A; HNF4A; HNF1B; INS; INSR; KCNJ11; NEUROD1; PDX1; PLIN1; RFX6; TRMT10A; WFS1*	307 (18%), 13	124 *GCK*, 63 *HNF1A*, 35 *HNF4A*, 18 *HNF1B*, 8 *ABCC8*, 1 *KCNJ11*, 4 *INS*, 7 *RFX6*, 5 *NEUROD1*, 7 *PDX1*, 24 m.3243A>G, 9 *WFS1*, 2 *INSR*
Singh, 2006 UK [27]	230 adults with type 2 DM and a negative m.3243A>G result using PCR-RFLP	Genotyping (TaqMan); 1	m.3243A>G	0 (0%), 0	
Støy, 2008 USA [28]	77 neonates with NDM	Sanger sequencing and MS-MLPA; 4	6q24*; ABCC8; INS; KCNJ11*	23 (30%), 3	14 *KCNJ11*, 7 *INS*, 2 6q24
Tosur, 2021 USA [29]	10 children with suspected MODY	targeted exome sequencing (gene panel); 70	*ABCC8; AGPAT2; AIRE; AKT2; APPL1; BLK; BSCL2; CDKN1C; CISD2; CEL; COQ2; COQ9; CP; CTLA4; DCAF17; DNAJC3; EIF2AK3;*	2 (20%), 2	1 *INS*, 1 *RFX6*

			EIF2S3; FOXP3; GATA4; GATA6; GCK; GLIS3; GLUD1; HADH; HNF1A; HNF4A; HNF1B; IER3IP1; IL2RA; INS; INSR; ITCH; KCNJ11; KLF11; LMNA; LPL; LRBA; MNX1; NEUROD1; NEUROG3; NKX2; NKX2-2; m.3243A>G; *PAX4; PAX6; PCBD1; PDX1; PIK3R1; PLIN1; POLD1; PPARG; PTF1A; RFX6; SIRT1; SLC2A2; SLC19A2; SLC29A3; STAT1; STAT3; STAT5B; TNFAIP3; TRMT10A; WFS1; ZBTB20; ZFP57*		
Weedon, 2014 UK [30]	22 neonateswith NDM	targeted genome sequencing (homozygosity mapping); 1	*PTF1A*	10 (48%), 1	10 *PTF1A*
Yan, 2014 China [31]	57 adults with suspected MIDD	Sanger sequencing and genotyping by Pyrosequencing, RFLP and HRM; 1	m.3243A>G	47 (83%), 1	47 m.3243A>G
Zmysłowska, 2022 Poland [32]	542 children with suspected MODY	tNGS (RNA baits); 35	*ABCC8; AIRE; APPL1; BLK; CISD2; CEL; EIF2AK3; FOXP3; GATA4; GATA6; GCK; GCKR; GLIS3; GLUD1; HADH; HNF1A; HNF4A; HNF1B; INS; ISL1; KCNJ11; KLF11; MAFA; MAFB; MNX1; NEUROD1; NEUROG3; NKX2-2; NKX6-1; PAX4; PAX6; PDX1; PTF1A; RFX6; WFS1*	198 (37%), 11	*GCK* 148, *HNF1A* 19, *HNF4A* 5, *HNF1B* 6, *ABCC8* 2, *KCNJ11* 8, *RFX6* 1, *PDX1* 5, *WFS1* 1, *MAFA* 1, *APPL1* 2

Supplementary References

1. Abbasi, F., et al., *A Genotype-First Approach for Clinical and Genetic Evaluation of Wolcott-Rallison Syndrome in a Large Cohort of Iranian Children With Neonatal Diabetes.* Can J Diabetes, 2018. **42**(3): p. 272-275.
2. Al Senani, A., et al., *Genetic mutations associated with neonatal diabetes mellitus in Omani patients.* J Pediatr Endocrinol Metab, 2018. **31**(2): p. 195-204.
3. Al-Kandari, H., et al., *Identification of Maturity-Onset-Diabetes of the Young (MODY) mutations in a country where diabetes is endemic.* Sci Rep, 2021. **11**(1): p. 16060.
4. Al-Khawaga, S., et al., *The clinical and genetic characteristics of permanent neonatal diabetes (PNDM) in the state of Qatar.* Mol Genet Genomic Med, 2019. **7**(10): p. e00753.
5. Alkorta-Aranburu, G., et al., *Phenotypic heterogeneity in monogenic diabetes: the clinical and diagnostic utility of a gene panel-based next-generation sequencing approach.* Mol Genet Metab, 2014. **113**(4): p. 315-320.
6. Alkorta-Aranburu, G., et al., *Improved molecular diagnosis of patients with neonatal diabetes using a combined next-generation sequencing and MS-MLPA approach.* J Pediatr Endocrinol Metab, 2016. **29**(5): p. 523-31.
7. Ang, S.F., et al., *A preliminary study to evaluate the strategy of combining clinical criteria and next generation sequencing (NGS) for the identification of monogenic diabetes among multi-ethnic Asians.* Diabetes Res Clin Pract, 2016. **119**: p. 13-22.
8. Anik, A., et al., *Molecular diagnosis of maturity-onset diabetes of the young (MODY) in Turkish children by using targeted next-generation sequencing.* J Pediatr Endocrinol Metab, 2015. **28**(11-12): p. 1265-71.
9. Antosik, K., et al., *Single patient in GCK-MODY family successfully re-diagnosed into GCK-PNDM through targeted next-generation sequencing technology.* Acta Diabetol, 2016. **53**(2): p. 337-8.
10. Ateş, E.A., et al., *Genetic and Clinical Characterization of Patients with Maturity-Onset of Diabetes of the Young (MODY): Identification of Novel Variations.* Balkan Med J, 2021. **38**(5): p. 272-277.
11. Bansal, V., et al., *Spectrum of mutations in monogenic diabetes genes identified from high-throughput DNA sequencing of 6888 individuals.* BMC Med, 2017. **15**(1): p. 213.
12. Berberich, A.J., et al., *Simplifying Detection of Copy-Number Variations in Maturity-Onset Diabetes of the Young.* Can J Diabetes, 2021. **45**(1): p. 71-77.
13. Carette, C., et al., *Familial young-onset forms of diabetes related to HNF4A and rare HNF1A molecular aetiologies.* Diabet Med, 2010. **27**(12): p. 1454-8.
14. Caswell, R.C., et al., *Noninvasive Fetal Genotyping by Droplet Digital PCR to Identify Maternally Inherited Monogenic Diabetes Variants.* Clin Chem, 2020. **66**(7): p. 958-965.
15. Chambers, C., et al., *Characteristics of maturity onset diabetes of the young in a large diabetes center.* Pediatr Diabetes, 2016. **17**(5): p. 360-7.
16. Colclough, K., et al., *Syndromic Monogenic Diabetes Genes Should Be Tested in Patients With a Clinical Suspicion of Maturity-Onset Diabetes of the Young.* Diabetes, 2022. **71**(3): p. 530-537.

17. De Franco, E., et al., *The effect of early, comprehensive genomic testing on clinical care in neonatal diabetes: an international cohort study.* Lancet, 2015. **386**(9997): p. 957-63.
18. Donath, X., et al., *Next-generation sequencing identifies monogenic diabetes in 16% of patients with late adolescence/adult-onset diabetes selected on a clinical basis: a cross-sectional analysis.* BMC Med, 2019. **17**(1): p. 132.
19. Ellard, S., et al., *Improved genetic testing for monogenic diabetes using targeted next-generation sequencing.* Diabetologia, 2013. **56**(9): p. 1958-63.
20. Ellard, S., et al., *Partial and whole gene deletion mutations of the GCK and HNF1A genes in maturity-onset diabetes of the young.* Diabetologia, 2007. **50**(11): p. 2313-7.
21. Johansson, S., et al., *Exome sequencing and genetic testing for MODY.* PLoS One, 2012. **7**(5): p. e38050.
22. Laimon, W., et al., *Genetic and clinical heterogeneity of permanent neonatal diabetes mellitus: a single tertiary centre experience.* Acta Diabetol, 2021. **58**(12): p. 1689-1700.
23. Patel, K.A., et al., *Systematic genetic testing for recessively inherited monogenic diabetes: a cross-sectional study in paediatric diabetes clinics.* Diabetologia, 2022. **65**(2): p. 336-342.
24. Patouni, K., et al., *A case of digenic maturity onset diabetes of the young with heterozygous variants in both HNF1Alpha and HNF1Beta genes.* Eur J Med Genet, 2021. **64**(9): p. 104264.
25. Pruhova, S., et al., *Glucokinase diabetes in 103 families from a country-based study in the Czech Republic: geographically restricted distribution of two prevalent GCK mutations.* Pediatr Diabetes, 2010. **11**(8): p. 529-35.
26. Saint-Martin, C., et al., *Gene Panel Sequencing of Patients With Monogenic Diabetes Brings to Light Genes Typically Associated With Syndromic Presentations.* Diabetes, 2022. **71**(3): p. 578-584.
27. Singh, R., et al., *Rapid and sensitive real-time polymerase chain reaction method for detection and quantification of 3243A>G mitochondrial point mutation.* J Mol Diagn, 2006. **8**(2): p. 225-30.
28. Stoy, J., et al., *Diagnosis and treatment of neonatal diabetes: a United States experience.* Pediatr Diabetes, 2008. **9**(5): p. 450-9.
29. Tosur, M., et al., *Exome sequencing in children with clinically suspected maturity-onset diabetes of the young.* Pediatr Diabetes, 2021. **22**(7): p. 960-968.
30. Weedon, M.N., et al., *Recessive mutations in a distal PTF1A enhancer cause isolated pancreatic agenesis.* Nat Genet, 2014. **46**(1): p. 61-64.
31. Yan, J.B., et al., *Pyrosequencing is an accurate and reliable method for the analysis of heteroplasmy of the A3243G mutation in patients with mitochondrial diabetes.* J Mol Diagn, 2014. **16**(4): p. 431-9.
32. Zmyslowska, A., et al., *Next- generation sequencing is an effective method for diagnosing patients with different forms of monogenic diabetes.* Diabetes Res Clin Pract, 2022. **183**: p. 109154.

Description of Supplementary Data Sets

Supplementary Data Set 1: Complete set of papers extracted for question 1. Who to test for monogenic diabetes.

Supplementary Data Set 2 : Complete set of papers extracted for question 2. How to test for monogenic diabetes
