# Supplementary material for: The use of precision diagnostics for monogenic diabetes: a systematic review and expert opinion
Source: Commun Med (Lond). 2023 Oct 5;3:136. doi: 10.1038/s43856-023-00369-8 (PMC10550998; doi:10.1038/s43856-023-00369-8)
Supplement: Supplementary file 4 — Description of Additional Supplementary Files [file 43856_2023_369_MOESM4_ESM.pdf]

Description of Additional Supplementary Files

File name: Supplementary Data 1

Description: Complete set of papers extracted for question 1. Who to test for monogenic diabetes.

File name: Supplementary Data 2

Description: Complete set of papers extracted for question 2. How to test for monogenic diabetes
